# Supplementary material for: High-Density Lipoprotein Changes in Alzheimer’s Disease Are APOE Genotype-Specific
Source: Biomedicines. 2022 Jun 24;10(7):1495. doi: 10.3390/biomedicines10071495 (PMC9312991; doi:10.3390/biomedicines10071495)
Supplement: Supplementary file 1 [file biomedicines-10-01495-s001.zip › biomedicines-1758344-supplementary.pdf]

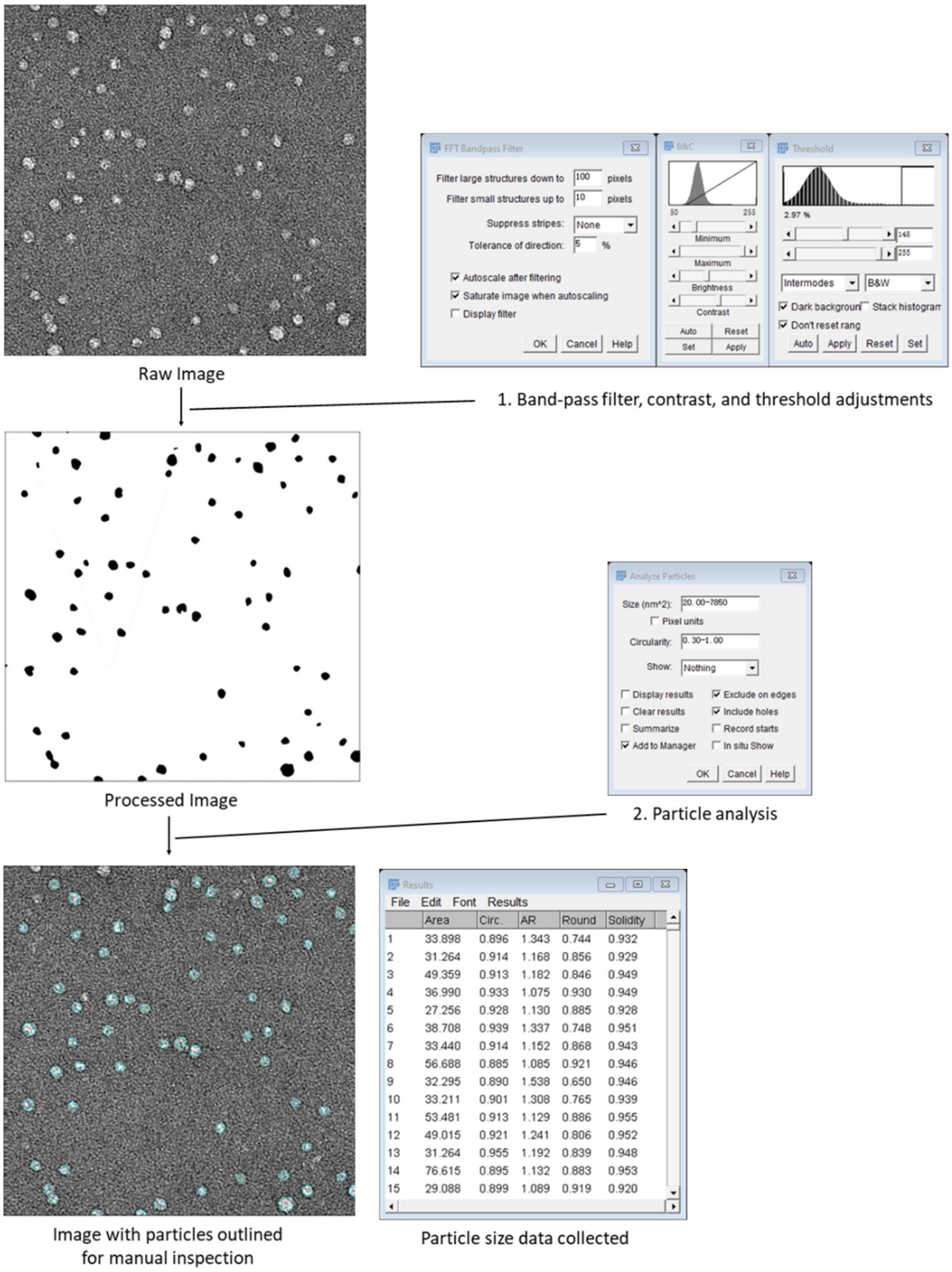

	Area	Circ.	AR	Round	Solidity
1	33.898	0.896	1.343	0.744	0.932
2	31.264	0.914	1.168	0.856	0.929
3	49.359	0.913	1.182	0.846	0.949
4	36.990	0.933	1.075	0.930	0.949
5	27.256	0.928	1.130	0.885	0.928
6	38.708	0.939	1.337	0.748	0.951
7	33.440	0.914	1.152	0.868	0.943
8	56.688	0.885	1.085	0.921	0.946
9	32.295	0.890	1.538	0.650	0.946
10	33.211	0.901	1.308	0.765	0.939
11	53.481	0.913	1.129	0.886	0.955
12	49.015	0.921	1.241	0.806	0.952
13	31.264	0.955	1.192	0.839	0.948
14	76.615	0.895	1.132	0.883	0.953
15	29.088	0.899	1.089	0.919	0.920

Figure S1. Schematic working flow for particle selection and size analysis on the sample TEM images. The "Bandpass Filter" function was called using the following parameters: "filter large = 100, filter small = 10, suppress = None, tolerance = 5, and autoscale saturate" parameters. The contrast of the cleaned micrograph was set to "min = 50, max = 205". The threshold of the micrographs was set using the "Intermodes dark" option. Particle analysis function was used with the following parameters: "size = 20–7850, circularity = 0.30–1.00, display, exclude, include, and add". Particles that were captured were then outlined onto the original TEM micrograph and were checked manually for accuracy.

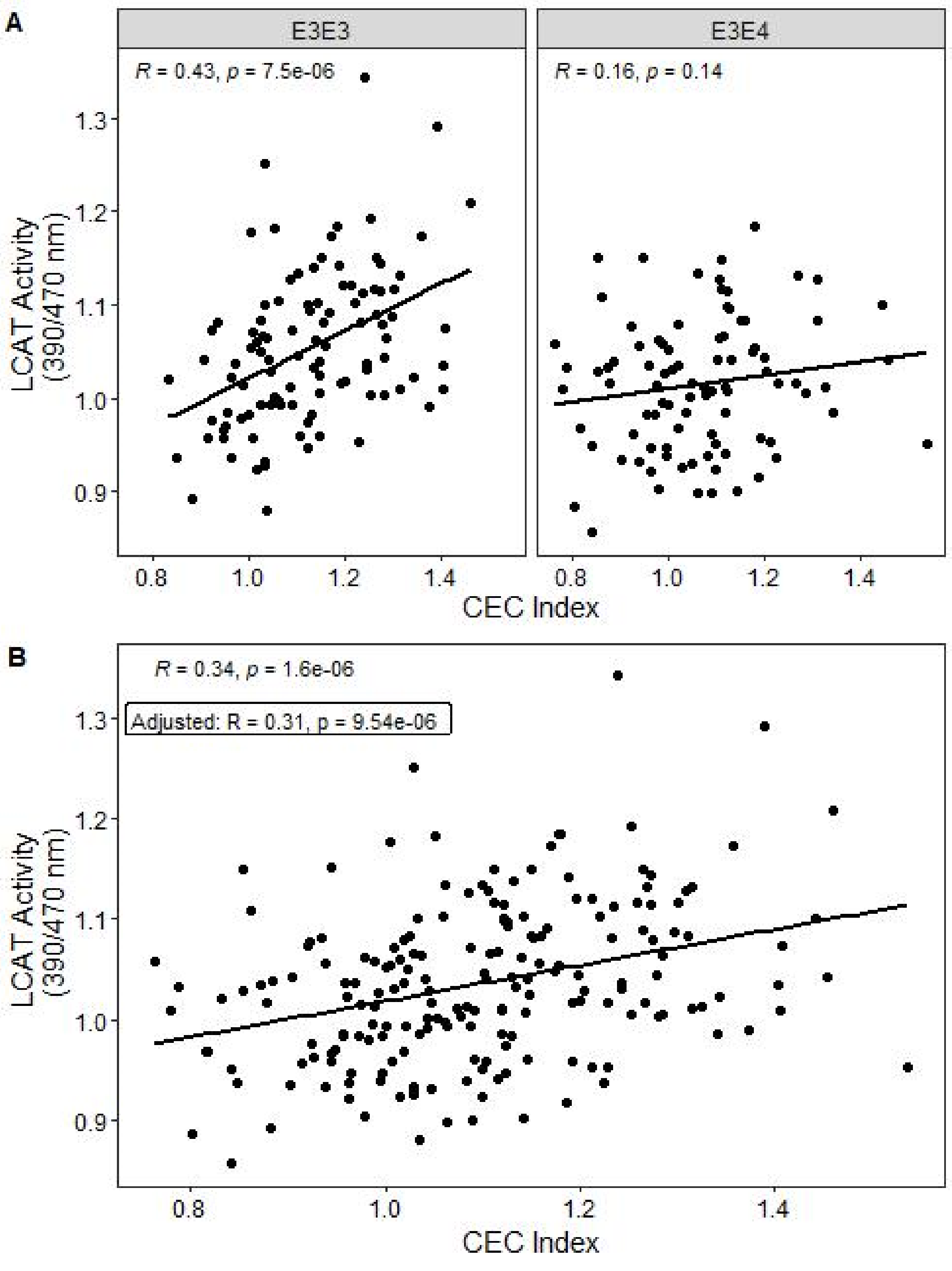

Figure S2. Spearman's correlation between HDL CEC and LCAT activity. (**A**) HDL LCAT activity and CEC index by the *APOE* genotype. (**B**) Spearman's correlation adjusting for the *APOE* genotype between the HDL LCAT activity and CEC index. CEC = cholesterol efflux capacity, HDL = high-density lipoproteins; LCAT= lecithin cholesterol acyltransferase.
